# Subclinical left ventricular diastolic dysfunction and incident type 2 diabetes risk: the Korean Genome and Epidemiology Study

**DOI:** 10.1186/s12933-017-0519-5

**Published:** 2017-03-14

**Authors:** Juri Park, Jin-Seok Kim, Seong Hwan Kim, Sunwon Kim, Sang Yup Lim, Hong-Euy Lim, Goo-Yeong Cho, Ki-Chul Sung, Jang-Young Kim, Inkyung Baik, Kwang Kon Koh, Jung Bok Lee, Seung Ku Lee, Chol Shin

**Affiliations:** 1 Department of Endocrinology, Hallym University Kangdong Sacred Heart Hospital, Seoul, South Korea; 2 Department of Cardiology, Korea University Ansan Hospital, Ansan, South Korea; 3 Cardiovascular Center, Korea University Guro Hospital, Seoul, South Korea; 4 Department of Cardiology, Seoul National University Bundang Hospital, Seongnam, South Korea; 5 Department of Cardiology, Kangbuk Samsung Hospital, Seoul, South Korea; 6 Department of Cardiology, Wonju College of Medicine, Yonsei University, Wonju, South Korea; 7 Department of Foods and Nutrition, Kookmin University, Seoul, South Korea; 8 Department of Cardiology, Gachon University Gil Medical Center, Incheon, South Korea; 9 Department of Clinical Epidemiology and Biostatistics, Asan Medical Center, Seoul, South Korea; 10 Institute of Human Genomic Study, Korea University Ansan Hospital, Ansan, South Korea; 11 Division of Cardiology, Department of Internal Medicine, Korea University Ansan Hospital, 123, Jeokgeum-ro, Danwon-gu, Ansan, Gyeonggi-do 15355 South Korea

**Keywords:** Left ventricle, Diastolic dysfunction, Tissue Doppler echocardiography, Type 2 diabetes, Cohort

## Abstract

**Background:**

Subclinical left ventricular (LV) diastolic dysfunction in type 2 diabetes (T2D) is a common finding and represents an early sign of diabetic cardiomyopathy. However, the relationship between LV diastolic dysfunction and the incident T2D has not been previously studied.

**Methods:**

A total of 1817 non-diabetic participants (mean age, 54 years; 48% men) from the Korean Genome and Epidemiology Study who were free of cardiovascular disease were studied. LV structure and function were assessed by conventional echocardiography and tissue Doppler imaging. Subclinical LV diastolic dysfunction was defined using age-specific cutoff limits for early diastolic (Em) velocity, mitral E/Em ratio, and left atrial volume index.

**Results:**

During the 6-year follow-up period, 273 participants (15%) developed T2D. Participants with incident T2D had greater LV mass index (86.7 ± 16.4 vs. 91.2 ± 17.0 g/m^2^), worse diastolic function, reflected by lower Em velocity (7.67 ± 1.80 vs. 7.47 ± 1.70) and higher E/Em ratio (9.19 ± 2.55 vs. 10.23 ± 3.00), and higher prevalence of LV diastolic dysfunction (34.6 vs. 54.2%), compared with those who did not develop T2D (all *P* < 0.001). In a multivariate logistic regression model, lower Em velocity (odd ratio [OR], 0.867; 95% confidence interval [CI] 0.786–0.957) and the presence of LV diastolic dysfunction (OR, 1.617; 95% CI 1.191–2.196) were associated with the development of T2D, after adjusting for potential confounding factors.

**Conclusions:**

In a community-based cohort, the presence of subclinical LV diastolic dysfunction was a predictor of the progression to T2D. These data suggest that the echocardiographic assessment of LV diastolic function may be helpful in identifying non-diabetic subjects at risk of incident T2D.

## Background

Recent advances in cardiovascular imaging technology have facilitated the detection of underlying subclinical target organ damage, which is useful for both the prediction of future cardiovascular disease (CVD) and risk stratification even in asymptomatic individuals with type 2 diabetes (T2D) [1, 2]. From a large body of experimental and clinical studies, it is already recognized that T2D is associated with various types of subclinical target organ damage resulting in an elevated risk of CVD events [3–5]. Moreover, there is growing evidence that a prediabetic status, such as impaired fasting glucose and impaired glucose tolerance, is also related to subclinical target organ damage when compared to a group with normal glucose metabolism (NGM) [6, 7].

Among the various forms of preclinical target organ damage observed in T2D, impaired left ventricular (LV) diastolic function, assessed using the combination of conventional and tissue Doppler imaging (TDI) echocardiography, is known to be an early sign of diabetic cardiomyopathy, even in the presence of normal LV systolic function [8]. Indeed, according to the typical sequence of the occurrence of target organ damage and T2D as indicated by the traditional CV continuum, T2D should antedate the development of subclinical target organ damage, such as LV diastolic dysfunction. However, considering recent studies and the available data on the association between prediabetes and an increased risk of CV events [9, 10], the presence of preclinical target organ damage in prediabetic patients seems to play an important role similar to that of T2D. In addition, in a recent study, the presence of subclinical target organ damage, including LV hypertrophy and carotid atherosclerosis, was shown to be a significant predictor of the development of new onset diabetes in hypertensive patients, independent of traditional CV risk factors [2]. However, it is still unclear whether the presence of asymptomatic target organ damage could help to identify sub-populations of non-diabetic individuals who are at increased risk of incident T2D. Accordingly, the present analysis was designed to evaluate the prospective relation between the presence of baseline LV diastolic dysfunction and the development of T2D in the general population.

## Methods

### Study population

Study subjects were recruited from an ongoing population-based Ansan cohort embedded in the Korean Genome Epidemiology Study, as previously described in detail [11]. The baseline cohort population (cycle 1) comprised 5020 members and has been followed biennially. This 6-year follow-up study (cycles 4–7) included the 3255 individuals who participated in the fourth cycle of the 2-year follow-up study (cycle 4) from March 12, 2007 to April 15, 2009 since the first echocardiographic study was performed at examination cycle 4. Those with known T2D and unavailable data on baseline glucose tolerance results were excluded at baseline (n = 721). Among 2,534 participants, we excluded if they had incomplete echocardiography data (n = 302); known CVD including previous history of myocardial infarction, coronary revascularization, angina, congestive heart failure, stroke, congenital heart disease, cardiomyopathy, significant valvular heart disease, arrhythmia, and an ejection fraction <50% (n = 26); or a serum creatinine level ≥2.0 mg/dL (n = 3). Additionally, non-diabetic participants who did not take part in the last visit (cycle 7) were also excluded (n = 518), leaving a total of 1817 subjects for the analysis.

The protocol of the study was approved by the Human Subjects Review Committee at the Korea University Ansan Hospital and all participants gave written informed consent.

### Measurements and definitions

Clinical information on demographics, CV risk factors, and medical history was obtained using interviewer-administered questionnaires. Family history of diabetes was defined as having a first-degree relative with diabetes. Height (cm) and body weight (kg) were measured and body mass index (BMI, kg/m^2^) was calculated. Subjects with BMI ≥23 kg/m^2^ were regarded as overweight according to Asian criteria [12]. Blood pressure was measured according to a standardized protocol using a mercury sphygmomanometer. According to the current guidelines, hypertension was defined as a systolic blood pressure ≥140 mmHg and/or diastolic blood pressure ≥90 mmHg and/or use of antihypertensive medication. After an at least an 8- to 14-h overnight fast, blood samples were collected for the measurement of serum total cholesterol, high-density lipoprotein (HDL) cholesterol, triglycerides (TG), fasting plasma glucose, fasting insulin, HbA1c, serum creatinine, and high-sensitivity C-reactive protein (hsCRP). The homeostasis model assessment insulin resistance index (HOMA-IR) was calculated as fasting serum insulin (µU/mL) × fasting plasma glucose (mg/dL)/405. In order to assess glucose tolerance status, all participants without known T2D underwent a 2-h 75-g oral glucose tolerance test at inclusion (cycle 4) and then biennially during a 6-year period. The definitions of NGM, prediabetes, and T2D were made according to 2016 ADA criteria [1]. NGM was defined as the presence of the following: a fasting plasma glucose <100 mg/dL, a 2-h plasma glucose <140 mg/dL, and HbA1c <5.7%. Prediabetes was defined as having impaired fasting glucose (fasting plasma glucose of 100–125 mg/dL) and/or impaired glucose tolerance (glucose of 140–199 mg/dL on a 2-h 75-g oral glucose tolerance test) and/or HbA1c of 5.7–6.4%. Incident T2D was defined as a fasting plasma glucose ≥126 mg/dL, a 2-h postprandial plasma glucose ≥200 mg/dL, HbA1c ≥6.5% or self-reported current use of anti-diabetic drugs or insulin at any follow-up examination cycle.

### Echocardiography

All echocardiographic examinations were performed using the Vivid 7 system (GE Vingmed, Horton, Norway) with a 4-MHz transducer according to the current recommendations [13]. Cardiac chamber diameters and wall thickness were measured by M-mode echocardiography. The area-length method and Devereux formula were used to calculate the left atrial (LA) volume and LV mass, respectively. Both LA volume and LV mass were indexed to body surface area and expressed as LA volume index and LV mass index. LV ejection fraction measurement was obtained using the modified biplane Simpson’s method. Transmitral peak E and peak A diastolic velocities and early mitral flow deceleration time (DT) were recorded at the tips of the mitral valve leaflets in an apical 4-chamber view. Tissue Doppler imaging of both peak systolic (Sm) and peak early diastolic (Em) velocities was measured at the septal side of the mitral annulus. Subsequently, the mitral E/Em ratio was calculated as an index of LV diastolic filling pressure. Echocardiographic LV hypertrophy was defined as an LV mass index >95 g/m^2^ in women and >115 g/m^2^ in men. LV diastolic dysfunction was defined based on a reduced septal TDI Em velocity, septal E/Em ratio >15, or LA volume index ≥34 mL/m^2^ [14]. To define a reduced septal TDI Em velocity, an age-specific abnormal value for septal TDI Em velocity was calculated as a value greater than one standard deviation below the mean reference value adjusted for age, since the TDI Em velocity is highly dependent on age [4, 15].

### Statistical analysis

Baseline demographic and echocardiographic data were presented as means ± standard deviations for continuous variables or as percentages for categorical variables. Differences between groups stratified by incident T2D were assessed using the Student’s *t* test for continuous variables or the χ^2^ test for categorical variables. Additionally, univariate logistic regression analyses were used to estimate the odds ratios of known cardiometabolic risk factors and echocardiographic parameters for the development of T2D. Finally, a multivariate logistic regression model tested the predictive value of each of the echocardiographic variables and the presence of baseline LV diastolic dysfunction for progression to T2D using age, sex, BMI ≥23 kg/m^2^, baseline glucose metabolism (NGM vs. prediabetes), hypertension, statin therapy, family history of diabetes, smoking status, alcohol intake, HOMA-IR, total cholesterol, TG/HDL ratio, and hsCRP as potential confounders. Covariates for the multivariate model were selected on the basis of univariate analyses and known risk factors for diabetes proposed by the latest diabetes guidelines [1]. To examine the effect of baseline glucose status, we performed additional multivariate logistic regression analyses after stratifying participants into two groups: “NGM” and “prediabetes”. For the multiple hypotheses testing, we corrected P values using the Bonferroni method based on the raw P values of logistic regression.

A P value < 0.05 was considered significant for all analyses. SAS version 9.3 (SAS institute, Cary, NC, USA) was used for all analyses.

## Results

### Baseline demographic, metabolic, and echocardiographic characteristics

Table 1 shows the baseline characteristics of the study subjects after stratification by incident T2D. Among the 1817 subjects without known CVD, 788 (43.4%) participants had NGM and 1029 (56.6%) had prediabetes at baseline according to ADA recommendations. Over the 6-year of follow-up period, 273 (129 males and 144 females) new cases of T2D occurred and 244 participants with incident T2D were prediabetic at inclusion. Overall, the subjects who developed incident T2D subjects displayed significant disturbances in various metabolic profiles at baseline, compared to participants without incident T2D. However, groups did not differ with regard to gender, heart rate, alcohol consumption, total cholesterol, and serum creatinine.

**Table 1:** Baseline demographic and laboratory characteristics of the study participants developing or not developing type 2 diabetes at the follow-up

Variable	All participants (n = 1817)	Not developing type 2 diabetes (n = 1544)	Developing type 2 diabetes (n = 273)	*P* value
Age (years)	53.5 ± 6.7	53.1 ± 6.4	55.7 ± 7.5	<0.001
Male (%)	47.9	48.1	47.3	0.844
BMI (kg/m^2^)	24.5 ± 2.7	24.3 ± 2.6	25.4 ± 2.8	<0.001
Systolic BP (mmHg)	110.3 ± 13.5	109.4 ± 13.3	115.1 ± 13.6	<0.001
Diastolic BP (mmHg)	74.6 ± 9.5	74.3 ± 9.6	76.1 ± 9.1	0.002
Heart rate (bpm)	65.0 ± 6.9	64.9 ± 6.9	65.4 ± 6.9	0.318
Hypertension (%)	22.1	19.7	35.9	<0.001
Antihypertensive therapy (%)	16.8	14.5	29.7	<0.001
Fasting glucose (mg/dL)	90.9 ± 8.4	89.9 ± 7.8	96.3 ± 9.7	<0.001
2-h glucose (mg/dL)	134.3 ± 30.6	129.8 ± 28.4	160.0 ± 29.7	<0.001
Fasting insulin (µIU/mL)	8.70 ± 4.03	8.52 ± 3.99	9.71 ± 4.12	<0.001
HbA1c (%)	5.43 ± 0.35	5.39 ± 0.33	5.65 ± 0.38	<0.001
HOMA-IR	1.97 ± 0.98	1.91 ± 0.95	2.33 ± 1.06	<0.001
Family history of diabetes (%)	17.5	16.6	22.7	0.016
Glucose metabolism (%)				<0.001
NGM	43.4	49.2	10.6	
Prediabetes	56.6	50.8	89.4	
Current smoker (%)	13.5	14.3	9.2	0.021
Current alcohol drinker (%)	50.0	50.1	49.5	0.896
Total cholesterol (mg/dL)	202.5 ± 34.2	202.1 ± 34.5	204.8 ± 32.9	0.217
HDL-cholesterol (mg/dL)	45.5 ± 10.6	45.9 ± 10.6	43.5 ± 10.5	0.001
Triglycerides (mg/dL)	134.6 ± 83.0	129.6 ± 79.5	162.3 ± 96.1	<0.001
Statin therapy (%)	2.5	1.4	3.4	0.007
hsCRP (mg/L)	1.39 ± 4.17	1.28 ± 3.93	2.03 ± 5.31	0.006
Creatinine (mg/dL)	0.95 ± 0.15	0.95 ± 0.14	0.96 ± 0.16	0.282

*BMI* body mass index, *BP* blood pressure, *HDL* high-density lipoprotein, *HOMA*-*IR* homeostasis model assessment-insulin resistance, *hsCRP* high sensitivity C-reactive protein, *NGM* normal glucose metabolism

Table 2 shows that the participants developing T2D had higher baseline relative wall thickness, LV mass index, mitral inflow A velocity, DT, and E/Em ratio, with lower baseline mitral inflow E velocity, mitral inflow E/A ratio, and TDI Sm and Em velocities compared to those without incident T2D (all *P* < 0.05). The prevalence of LV hypertrophy and LV diastolic dysfunction was also higher in participants developing T2D (all *P* < 0.05). On the other hand, no significant differences in terms of LA volume index and LV ejection fraction were shown.

**Table 2:** Baseline echocardioraphic parameters of the study participants developing or not developing type 2 diabetes at the follow-up

Variable	Total (n = 1817)	Not developing type 2 diabetes (n = 1544)	Developing type 2 diabetes (n = 273)	*P* value
LA volume index (mL/m^2^)	26.2 ± 6.3	26.1 ± 6.3	26.7 ± 6.4	0.181
Relative wall thickness	0.37 ± 0.06	0.36 ± 0.06	0.39 ± 0.07	<0.001
LV mass (g)	150 ± 37	149 ± 37	158 ± 38	<0.001
LV mass index (g/m^2^)	87.3 ± 16.6	86.7 ± 16.4	91.2 ± 17.0	<0.001
LV hypertrophy (%)	13.9	13.1	18.7	0.017
LV ejection fraction (%)	65.0 ± 4.5	65.1 ± 4.5	64.7 ± 4.5	0.235
Mitral inflow velocity				
E, cm/s	0.68 ± 0.15	0.68 ± 0.15	0.66 ± 0.15	0.039
A, cm/s	0.63 ± 0.17	0.62 ± 0.17	0.70 ± 0.18	<0.001
E/A ratio	1.15 ± 0.36	1.17 ± 0.37	0.99 ± 0.30	<0.001
DT, ms	195 ± 47	195 ± 47	200 ± 47	0.091
Tissue Doppler imaging (TDI)				
TDI Sm velocity (cm/s)	7.68 ± 1.29	7.70 ± 1.29	7.47 ± 1.27	0.018
TDI Em velocity (cm/s)	7.57 ± 1.83	7.67 ± 1.80	7.47 ± 1.70	<0.001
E/Em ratio	9.31 ± 2.62	9.19 ± 2.55	10.23 ± 3.00	<0.001
LV diastolic dysfunction (%)	37.5	34.6	54.2	<0.001

*DT* deceleration time, *LA* left atrium, *LV* left ventricle

### Independent predictors of incident T2D

After 6 years, the development of T2D was associated with baseline age, excessive body weight, the presence of prediabetes and hypertension, statin therapy, current smoking, HbA1c, HOMA-IR, low HDL-cholesterol level, high TG level, hsCRP, and family history of diabetes in univariate analyses (all *P* < 0.05).

Table 3 shows that various echocardiographic LV structural and functional parameters except for LA volume index and the presence of LV hypertrophy and LV diastolic dysfunction were independent predictors for the development of T2D in univariate analyses. When each echocardiographic parameter was separately forced into a multivariate logistic regression model including age, sex, BMI ≥23 kg/m^2^, baseline glucose metabolism (NGM vs. prediabetes), hypertension, statin therapy, family history of diabetes, smoking status, alcohol intake, HOMA-IR, TG/HDL ratio, and hsCRP, only TDI Em velocity was inversely and independently associated with incident T2D. Similarly, the risk of 6-year incidence of T2D increased by 62% in the presence of baseline LV diastolic dysfunction, regardless of baseline glucose metabolism status (Table 3). None of the echocardiographic measurements of LV systolic function showed significant predictive effect.

**Table 3:** Baseline echocardiographic parameters associated with incident type 2 diabetes

Variables	Univariate		Multivariate^a^		
	OR (95% CI)	*P* value	OR (95% CI)	*P* value	*P* value^b^
LA volume index (mL/m^2^)	1.013 (0.993–1.034)	0.189	1.017 (0.995–1.040)	0.133	0.933
LV mass index (g/m^2^)	1.016 (1.008–1.024)	<0.001	1.009 (1.000–1.018)	0.040	0.283
LV hypertrophy (yes vs. no)	1.526 (1.088–2.141)	0.014	1.198 (0.806–1.780)	0.371	1.000
TDI Sm velocity (cm/s)	0.856 (0.772–0.949)	0.003	0.914 (0.816–1.024)	0.119	0.836
TDI Em velocity (cm/s)	0.742 (0.685–0.804)	<0.001	0.867 (0.786–0.957)	0.004	0.031
E/Em ratio	1.147 (1.094–1.201)	<0.001	1.076 (1.017–1.137)	0.010	0.071
LV diastolic dysfunction (yes vs. no)	2.239 (1.726–2.905)	<0.001	1.617 (1.191–2.196)	0.002	0.014

*BMI* body mass index, *CI* confidence interval, *HDL* high-density lipoprotein, *HOMA*-*IR* homeostasis model assessment-insulin resistance, *hsCRP* high sensitivity C-reactive protein, *LA* left atrium, *LV* left ventricle, *NGM* normal glucose metabolism, *OR* odds ratio, *TDI* tissue Doppler imaging, *TG* triglycerides

^a^Model was adjusted for age, sex, BMI ≥23 kg/m^2^, baseline glucose metabolism (NGM vs. prediabetes), hypertension, statin therapy, family history of diabetes, smoking status, alcohol intake, HOMA-IR, total cholesterol, TG/HDL ratio, and hsCRP. Each echocardiographic variable was tested separately in a multivariate model

^b^Corrected P values using Bonferroni method for multiple comparisons

The multivariate logistic regression analyses, adjusting for age, sex, BMI ≥23 kg/m^2^, fasting plasma glucose, 2-h plasma glucose, mean blood pressure, family history of diabetes, smoking status, alcohol intake, HOMA-IR, total cholesterol, TG/HDL ratio, and hsCRP, were also performed in a specific subpopulation stratified according to the baseline glucose metabolism (NGN vs. prediabetes). Prediabetic individuals were older, more frequent alcohol drinker, had higher baseline BMI, systolic and diastolic blood pressures, heart rate, total cholesterol, triglycerides, hsCRP, lower HDL-cholesterol, and more frequent family history of diabetes (all *P* < 0.05) than participants with NGM. In the prediabetic population, incident T2D was independently predicted by the presence of LV diastolic dysfunction, but any of the echocardiographic parameters, even with the presence of LV diastolic dysfunction, could not predict the development of T2D in subjects with NGM (Table 4).

**Table 4:** Echocardiographic predictors of incident type 2 diabetes in a subgroup stratified according to baseline glucose metabolism (NGM vs. prediabetes), adjusting for age, sex, BMI ≥23 kg/m^2^, fasting plasma glucose, 2-h plasma glucose, hypertension, statin therapy, family history of diabetes, smoking status, alcohol intake, HOMA-IR, total cholesterol, TG/HDL ratio, and hsCRP: multivariate logistic regression analyses

Variables	NGM (n = 788)			Prediabetes (n = 1029)		
	OR (95% CI)	*P* value	*P* value^a^	OR (95% CI)	*P* value	*P* value^a^
LA volume index (mL/m^2^)	1.037 (0.982–1.095)	0.190	1.000	1.021 (0.993–1.048)	0.140	1.000
LV mass index (g/m^2^)	0.999 (0.973–1.026)	0.948	1.000	1.014 (1.004–1.025)	0.009	0.126
LV hypertrophy (yes vs. no)	0.848 (0.268–2.683)	0.779	1.000	1.315 (0.833–2.074)	0.240	1.000
TDI Sm velocity (cm/s)	0.892 (0.633–1.257)	0.513	1.000	0.872 (0.764–0.995)	0.041	0.574
TDI Em velocity (cm/s)	0.958 (0.725–1.267)	0.765	1.000	0.864 (0.770–0.968)	0.012	0.168
E/Em ratio	1.078 (0.900–1.292)	0.416	1.000	1.064 (1.000–1.134)	0.051	0.714
LV diastolic dysfunction (yes vs. no)	1.000 (0.389–2.575)	0.999	1.000	1.906 (1.335–2.721)	<0.001	0.005

*BMI* body mass index, *CI* confidence interval, *HDL* high-density lipoprotein, *HOMA*-*IR* homeostasis model assessment-insulin resistance, *hsCRP* high sensitivity C-reactive protein, *LV* left ventricle, *NGM* normal glucose metabolism, *OR* odds ratio, *TDI* tissue Doppler imaging, *TG* triglycerides

^a^Corrected P values using Bonferroni method for multiple comparisons

In a subgroup analysis, we conducted post hoc power analyses using GPower software with power at 0.80 and alpha = 0.05 (two-tailed) to estimate the sample sizes for LV diastolic parameters and the presence of LV diastolic function. This showed us that the sample sizes in NGM group would have to increase up to 14,132 and 311,264 for TDI Em velocity and LV diastolic dysfunction, respectively, to reach statistical significance at the 0.05 level. Therefore, it is unlikely that our negative results in subjects with NGM can be attributed to a limited sample size.

## Discussion

We found that the presence of LV diastolic dysfunction, one of the various types of target organ damage induced by overt T2D, was a significant predictor of incident T2D, independent of various cardiometabolic profiles. In addition, LV diastolic markers such as mitral inflow E/A ratio, TDI Em velocity, and E/Em ratio as continuous variables were also associated with an increased incidence of T2D. This finding was confirmed in a subgroup of prediabetes, whereas neither LV diastolic parameters nor the existence of LV diastolic dysfunction were associated with the risk of incident T2D in participants with NGM.

### Association of CV risk factors with target organ damage

According to the CV continuum theory, a clear temporal relationship exists whereby various CV risk factors, such as hypertension and T2D, generally precede the development of target organ damage. However, recent literatures have reported that increased LV mass and arterial stiffness are predictors of incident hypertension in both normotensive and prehypertensive individuals [16, 17]. Moreover, our previous findings demonstrated that the clustering of target organ damage types, including LV hypertrophy, LV diastolic dysfunction, carotid atherosclerosis, and arterial stiffness, substantially increases the risk of developing hypertension in the non-hypertensive population, irrespective of baseline BP category and obesity status [18]. Similar to previous reports regarding the temporal correlation between incident hypertension and preclinical types of target organ damage, Izzo et al. [2] reported that the presence of target organ damage, such as LV hypertrophy and carotid atherosclerosis, is a significant predictor of new-onset T2D in a population of treated hypertensive patients. Thus, these findings, which are different from the traditional concept of CV continuum, suggest that the presence of several types of asymptomatic target organ damage may be helpful in predicting incident T2D and hypertension.

### Association of glucose metabolism with cardiac function

To our knowledge, no epidemiological study has specifically linked preclinical LV diastolic dysfunction to incident T2D. Instead, previous cross-sectional studies have shown that subtle abnormalities of LV systolic and/or diastolic function, which are key components of the progression to diabetic cardiomyopathy, are frequently detected in T2D [4, 19–23]. Although the exact causes leading to the LV changes in patients with T2D remain still unclear, the reduction of coronary flow reserve, metabolic abnormality, autonomic dysfunction, and myocardial fibrosis have been reported as possible mechanisms of subclinical myocardial damage [24]. Besides these data, subclinical LA structural and functional changes are also known to be common findings in patients with T2D [25–27]. Interestingly, Wang et al. reported that LA energy loss and deformation mechanics are already impaired even in T2D patients with normal LA size [27]. Overall, the duration of diabetes, diabetic complications, hypertriglycemia, obesity status, blood pressure, and glycemic control in asymptomatic patients with T2D were closely related to LA remodeling and LV diastolic dysfunction. However, according to recent observations, the high prevalence of subclinical LV systolic and/or diastolic dysfunction has been noted in prediabetic subjects compared to those with normal glucose metabolism, although prediabetes have intermediate values for various indices of LV systolic and diastolic function between normal and diabetic states [6, 28]. In addition, an association of prediabetes with other types of target organ damage, such as carotid atherosclerosis and arterial stiffness, has been reported, independent of other potential metabolic risk factors [7, 29]. As a result, although the clinical significance of early subclinical changes in the CV system observed in the prediabetic population has been in question in previous cross-sectional studies, the prognostic impact of LV diastolic dysfunction in prediabetes from our findings supports the hypothesis that preclinical target organ damage may be predictive of T2D development.

Although the exact mechanism linking the impairment of LV diastolic function to incident T2D in prediabetic individuals could not be determined in the current analysis, it can be hypothesized that the disturbance of myocardial insulin signaling plays an important role in its pathogenesis [30]. Similar to the relationship between insulin resistance and its associated CV complications in overt T2D, recent studies have indicated that higher levels of insulin resistance were related to the impairment of cardiac and vascular function compared to those with lower insulin resistance in a non-diabetic population [31, 32]. Therefore, considering that insulin resistance is a characteristic feature of T2D, and that higher levels of HOMA index were associated with incident T2D, our suggestion that prediabetic subjects with LV diastolic dysfunction are at higher risk of developing overt T2D appears plausible.

### Limitations

Our study has several limitations. Firstly, not all of the parameters for the precise evaluation of LV diastolic function were measured. Therefore, although previous studies reported a wide range of the prevalence of LV diastolic dysfunction ranging from 11.1 to 34.7% in the general population [33], the prevalence (37.5%) of LV diastolic dysfunction as determined in our study seems somewhat high. Nevertheless, given that each measured value for assessing LV diastolic function was an independent predictor of incident T2D, the arbitrary definition of LV diastolic dysfunction used in our study seems acceptable. Another limitation is that since our cohort members were all Korean, so to apply our findings to other ethnicities may not be valid. Thus, additional research is required.

## Conclusions

Left ventricular diastolic parameters and the presence of LV diastolic dysfunction were independent predictors of 6-year incident T2D in a population-based sample without overt CVD. These findings may have potential clinical implications for primary CV prevention because early identification of LV diastolic dysfunction could offer substantial opportunities to delay the development of T2D through more aggressive and preventive strategies, especially in the prediabetic population at high risk for developing T2D. Although the current guidelines do not recommend the routine use of screening tests to search for asymptomatic target organ damage in non-diabetic individuals, our study suggest that cardiac ultrasound may be a useful screening tool in a clinical practice. Future trials are needed to determine whether earlier and more aggressive intervention could prevent or delay the development of T2D in these individuals.

## Abbreviations

BMI: body mass index
CVD: cardiovascular disease
DT: deceleration time
HDL: high-density lipoprotein
HOMA-IR: homeostasis model assessment insulin resistance index
hsCRP: high-sensitivity C-reactive protein
LA: left atrium
LV: left ventricle
NGM: normal glucose metabolism
TDI: tissue Doppler imaging
TG: triglycerides
T2D: type 2 diabetes
